# Using Machine Learning to Uncover Hidden Heterogeneities in Survey Data

**DOI:** 10.1038/s41598-019-51862-x

**Published:** 2019-11-05

**Authors:** Christina M. Ramirez, Marisa A. Abrajano, R. Michael Alvarez

**Affiliations:** 1 Department of Biostatistics, UCLA Fielding School of Public Health, UCLA, Los Angeles, CA 90095-1772 USA; 2 Department of Political Science, University of California, San Diego, La Jolla, CA 92093-0521 USA; 3 Division of Humanities and Social Sciences, California Institute of Technology, Pasadena, CA 91125 USA

**Keywords:** Preventive medicine, Health policy, Population screening

## Abstract

Survey responses in public health surveys are heterogeneous. The quality of a respondent’s answers depends on many factors, including cognitive abilities, interview context, and whether the interview is in person or self-administered. A largely unexplored issue is how the language used for public health survey interviews is associated with the survey response. We introduce a machine learning approach, Fuzzy Forests, which we use for model selection. We use the 2013 California Health Interview Survey (CHIS) as our training sample and the 2014 CHIS as the test sample. We found that non-English language survey responses differ substantially from English responses in reported health outcomes. We also found heterogeneity among the Asian languages suggesting that caution should be used when interpreting results that compare across these languages. The 2013 Fuzzy Forests model also correctly predicted 86% of good health outcomes using 2014 data as the test set. We show that the Fuzzy Forests methodology is potentially useful for screening for and understanding other types of survey response heterogeneity. This is especially true in high-dimensional and complex surveys.

## Introduction

A growing body of research in the United States notes that a lack of English-language proficiency is associated with disparities in health care outcomes^1–3^. It is clear that in language communities, limited English proficiency carries widespread implications for public health outcomes across patients from different racial and ethnic backgrounds, and for different age cohorts. Given the potential ubiquity of public health outcome disparities in the U.S., it is critical that the way that language use is associated with health treatment and outcomes is adequately accounted for in analyses of public health surveys.

The typical public health survey, especially those that are conducted in-person or over the telephone (“interviewer-assisted”), can be thought of as a conversation between the subject and the interviewer^4,5^. Like any social interaction, the conversation about public health topics between subject and interviewer is susceptible to a variety of different contextual effects: environmental, inter-personal, and psychological. A male survey respondent might answer questions about his health or sexual activity differently when the interviewer is male, than if the interviewer is female; likewise, an African-American female survey respondent might provide different survey responses about her health or sexual activity to a white female interviewer than to an African-American female interviewer (see the review in^6^). Interview context can also matter when the survey does not involve an interviewer. In a self-completion survey, particularly those conducted online, the subject might complete the survey while engaged in other tasks, and not be as cognitively engaged in thinking about the survey questions as the researcher might desire^7^. Furthermore, how questions are framed, worded, and ordered are all associated with the quality and nature of the survey response^5,8^.

Heterogeneity in survey responses can take many forms, including differential response variability^9^ or a lack of measurement equivalence^10^. These same types of survey response issues will arise with respect to language. A growing body of research demonstrates the relationship between interview language and survey responses^11–14^. There are numerous ways in which the language of interview can affect a survey response. First, words, concepts, and framing of survey questions may take on different meanings when they are translated into various languages. Such variations can introduce heterogeneity into the survey response, and thus a respondent taking the survey in one language may respond quite differently to it than a respondent taking the survey in another language, as a result of these differences in meaning (subtle or not). Second, language may have a direct effect on individual attitudes, and thus a question asked to one respondent in Spanish might invoke a different cognitive or attitudinal process than for those asked the same question in English^14–16^. Finally, a survey questionnaire might be poorly translated, so that the meaning of questions or responses may be dissimilar across different versions of the survey instrument. All of these processes will introduce significant heterogeneity into the survey response, and if handled inappropriately, will result in misleading or biased inferences from these surveys.

Currently, survey methodologists lack a coherent theory of how language might be associated with attitudes or survey responses, despite mounting evidence that the language of interview is related to many survey responses^14^. Without strong theory to guide researchers who are concerned about language-of-interview effects in surveys, users of survey datasets do not know whether responses differ based on the language of the interview, nor how they may differ. We contend that researchers could use machine learning approaches to determine: (1) whether language-of-interview differences are prevalent in the survey data they are using; (2) how to specify those differences, and; (3) how those differences are associated with substantive inferences that might otherwise be ignored when researchers overlook language-of-interview effects. These issues are especially true when a large number of potential predictors exist or when these associations may be non-linear or interactive in nature.

Accounting for language differences is an especially important problem for studies using public health surveys. While Ponce *et al*.^17^ have documented the association between English-language proficiency and public health survey responses, the following questions remain unanswered: how does a researcher using a public health survey determine whether language differences in survey responses exist? And if such differences do exist, how they should be incorporated into survey design, analysis and modeling. The goal of this paper is to provide guidance to the first question, that we hope will then lead to future research to answer the second question.

While all of these artifacts and biases are well-documented in applied survey research, at present survey methodologists and users of public health survey data lack readily established tools to diagnose a survey dataset for these biases. Although tools that can correct survey datasets for these biases have been developed recently^18,19^, this is an area of active research. In this paper, we argue that a new machine learning technique, the Fuzzy Forests, is a helpful tool that can be used to screen for certain types of survey response biases and heterogeneities in survey data. Fuzzy Forests extends Random Forests and is suited for detecting important (and potentially problematic) heterogeneities in survey response data. Unlike the more familiar Random Forest, Fuzzy Forests works well for variable selection when the features or covariates in the model are highly intercorrelated (which is typically true of survey data). Fuzzy Forests creates a weighted correlation network that separates the predictors into modules of similar covariates which can be further explored to determine which are the most important covariates within each module that are contributing to the outcome of interest. Using this technique can possibly lend insights into the associations between the survey questions and the survey response.

In this paper, we examine an important question in public health surveys, the predictors of poor health responses. We use Fuzzy Forests to determine whether differences exists in response to assessment’s about one’s own health, and how those differences could be included in a statistical model. Specifically, we use survey responses from the publicly available 2013 and 2014 California Health Interview Survey (CHIS), which is the largest health survey to be conducted in the United States. As we mentioned earlier, our analysis focuses on the survey item that asks respondents to evaluate their health, with responses ranging from excellent to poor. Our Fuzzy Forests analysis reveals that interview language is an important predictor of reported poor health.

## Results

Supplementary Fig. 1 provides the results from the Fuzzy Forests analysis, where we show the relative feature or variable importance. We see that Fuzzy Forests, regardless of the module structure selected, found an interesting predictor in the 2013 CHIS data for overall general health: “INTVLANG”, or the interview language variable. Using various module structures, different values of *mtry*, and repeating the analysis with different seeds 1000 times, we found that interview language consistently emerged among the top 20 predictors, and in fact, was among a small handful of factors that strongly predicted overall general health. We therefore view this finding to be quite robust, and one with important implications for use of the CHIS survey data. We return to this point later on in the paper.

Note that stage 1 of the Fuzzy Forests algorithm creates a weighted correlation network of the predictor variables. This is an unsupervised learning step that does not take into consideration the outcome variable. Also, the correlation network is weighted such that we obtain approximate scale-free topology. Supplemental Fig. 2 presents the hierarchical clustering dendogram of the module eigenfactors. The module eigenfactors are obtained by the first principle component of each module matrix. Supplemental Tables 1–9 show the variable names and labels that constitute each module that was constructed such that we had approximate scale-free topology. The modules appear to be clustered by topic. Supplemental Fig. 3 shows the “modplot”, or the percent “important” by each module. Modplots can help design future surveys as they show which modules are important in predicting the outcome, and thus could be used to design shorter (and less costly) surveys. The modules can also be used to help understand the mechanisms behind the results. In this case the Brown (listed in Supplemental Table 3) and the Black modules (listed in Supplemental Table 1) had the highest percentage of features that were important in predicting the outcome. The Black module had variables related to disability, psychological distress and depression while the Brown module had variables that encompass languages spoken, English proficiency, food insecurity and welfare status.

**Figure 1:**
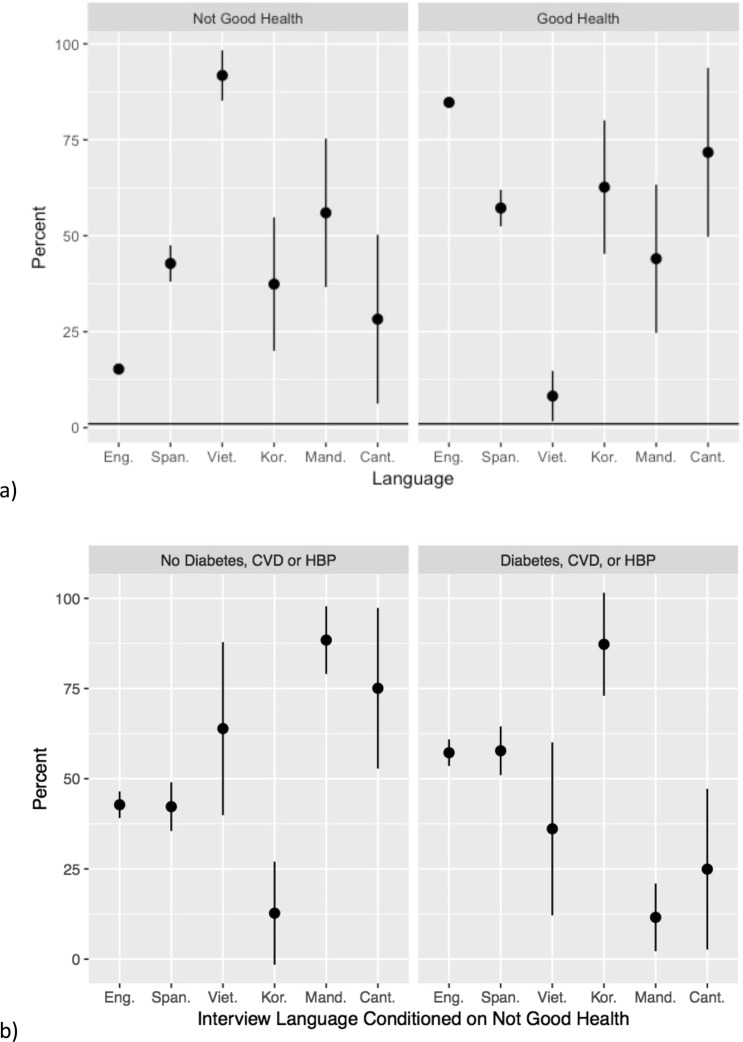
(**a**) Health outcomes by interview language. Eng-English, Span-Spanish, Viet-Vietnamese. Kor-Korean, Mand-Mandarin, Cant-Cantonese. Good health is defined as respondent who reported their general overall health was Excellent, Very Good or Good. Not Good health is defined as self-reported fair or poor overall general health. (**b**) Percent of subjects with chronic health conditions by interview language conditioned on subject reporting not good health. CVD: Cardiovascular Disease, HBP: High Blood Pressure.

Given these results, we conducted further analysis that examines the relationship between overall health status and language of interview in the 2013 CHIS data. Figure 1a presents the proportions of survey respondents who said they did not have good health (left panel), and the proportions who said they had good health (right panel), by language of survey interview. Note that there were too few respondents who completed the 2013 interview in Tagalog to include them in this analysis. As Fig. 1a clearly demonstrates, respondents who participated in the English-language survey reported distinct health outcomes, relative to those who were interviewed in another language. In particular, those respondents interviewed in Spanish, Vietnamese, or Mandarin were more likely than those who were interviewed in English to report bad health outcomes. Importantly, we also see substantial heterogeneity across the the Asian languages regarding their health outcomes, suggesting that analysts of these data may want to avoid combining responses for those taking the interview in different Asian languages.

Of course, this relationship could arise for numerous reasons, in particular because of differences in socioeconomic status, or other general difficulties in life circumstances, that might be associated with the language that these respondents are most comfortable using. For confirmatory analysis, we created an indicator variable for chronic conditions such as diabetes, cardiovascular disease and high blood pressure. Subjects were coded as having a chronic condition if they had any one or more of those conditions. Figure 1b shows the percent of subjects who had either diabetes, cardiovascular disease and/or high blood pressure stratified by interview language conditioned on stating that they did not have good health. Interestingly, with the exception of those taking the survey in Korean, respondents taking the survey in Asian languages were less likely to report that they had diabetes, cardiovascular disease or high blood pressure given that they stated they were not in good health.

**Figure 2:**
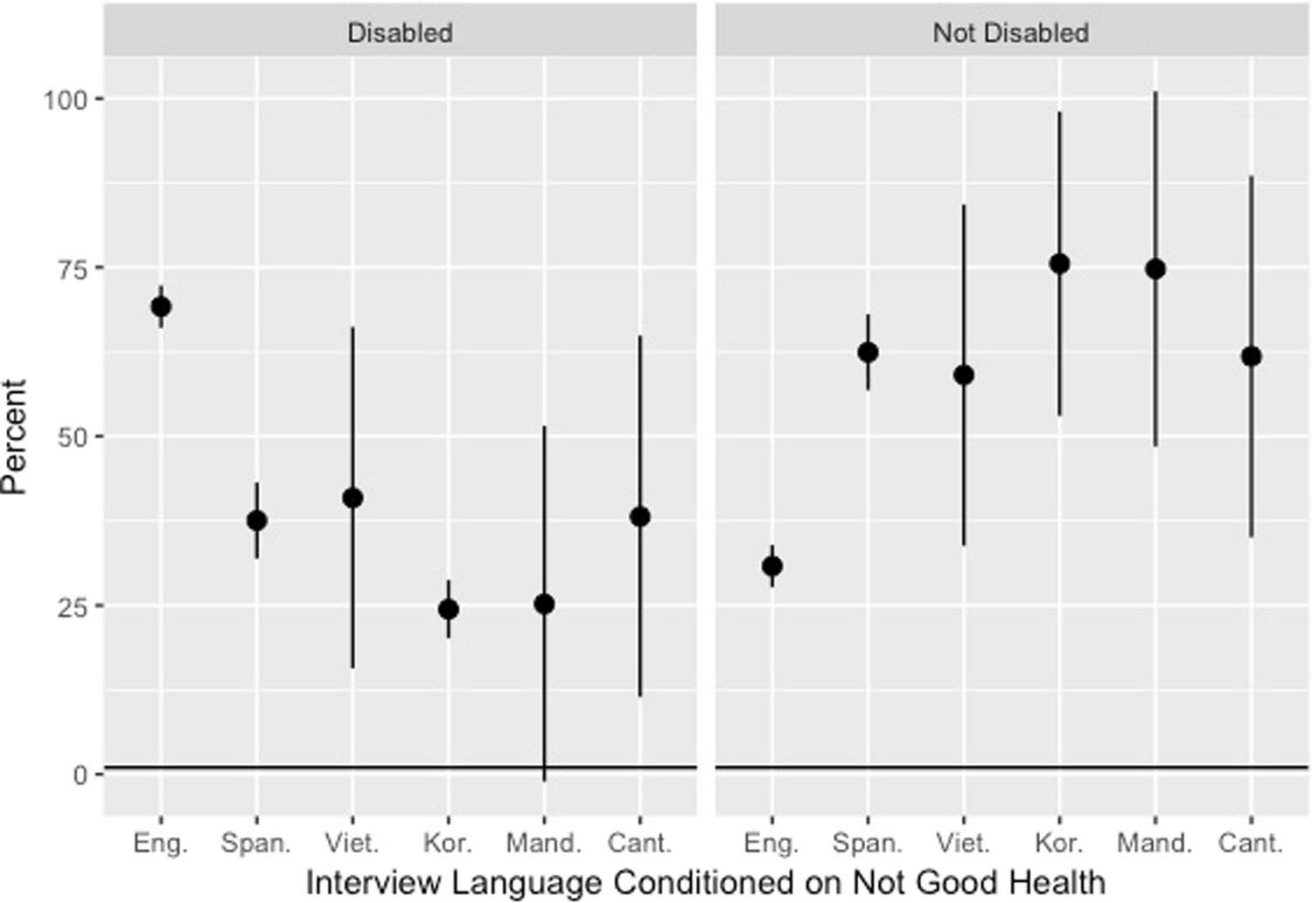
Percent of subjects who reported being disabled by interview language conditioned on not having good health.

Figure 2 shows the percent of subjects who reported bad health and also reported that they had a condition that rendered them disabled. Note that those who took the survey in English and reported that they did not have good health were more likely to report being disabled. These estimates once again demonstrate the existence of heterogeneity by interview language, as we see that important interview language differences arise in the reported disability and chronic conditions by health outcomes. As such, it is important to assess the association between interview language and these predictors.

We also performed a more traditional forward and backwards variable selection weighted logistic regression, as examples of what a researcher uncertain about which variables to include in a model might attempt. Techniques like forward and backward variable selection have been used in the public health literature, so they provide useful methods to compare with the performance of our Fuzzy Forests model (for examples from the public health literature see^20–23^). However, both approaches were very computationally intensive and time consuming, and worse, they produced inconsistent selection of variables. For example, forwards selection produced a model with 50 important covariates while backwards selection resulted in model with 209 covariates. In this case, importance was defined as having a *p*-value < 0.01. These findings did not come as a great surprise, since this is a known issue with these selection methods. However, while the overlap between the two methods in terms of variables selected were not exact, both methods found interview language to be important, further suggesting the salience of interview language was not due to random chance. We also note that identification required much less computational effort in our Fuzzy Forests model, relative to other estimation techniques.

**Figure 3:**
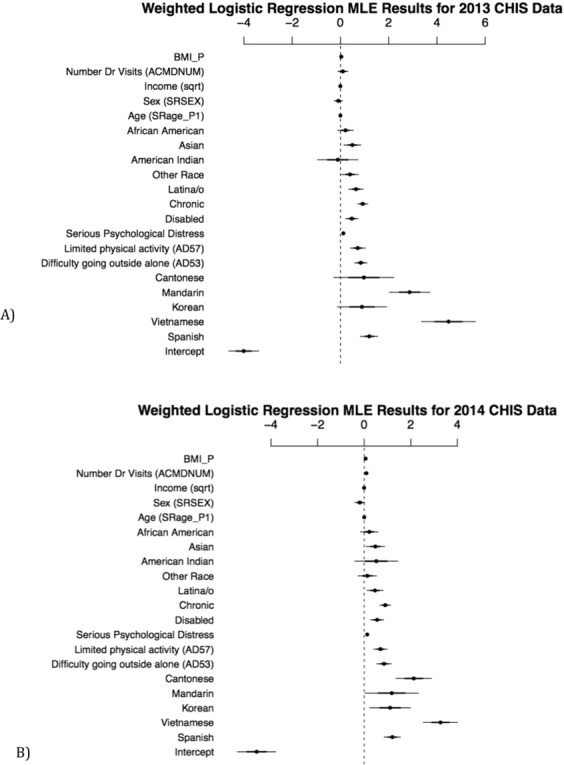
(**a**) Weighted Logistic Regression MLE parameter estimates and 95% confidence interval using 2013 CHIS data. (**b**) Weighted Logistic Regression MLE parameter estimates and 95% confidence interval using 2014 CHIS data.

Next, we conducted a two-stage model and estimated a survey-weighted logistic regression using the top predictors of the Fuzzy Forests controlling for age, income (as square root of income), gender identity, and ethnicity. Interview language still emerged as highly significant after controlling for potential confounders. These estimates provide us with even more confidence that it is an important predictor of reported overall general health. Figure 3a shows the parameter estimates, while Table 1 provides the odds ratios for the logistic regression estimates. Note that due to instability in the estimates from a sample size of 1, we deleted respondents taking the survey in Tagalog.

**Table 1:** Odds Ratios and 95% Wald Confidence Limits for 2013 CHIS Data.

Parameter	Label	Point Estimate	95% Wald Confidence Limit	
RACEHP2_P1	African American vs White	1.236	0.898	1.702
RACEHP2_P1	American Indian/Alaskan vs White	0.897	0.392	2.054
RACEHP2_P1	Asian vs White	1.643	1.160	2.327
RACEHP2_P1	Latino/a vs White	1.905	1.419	2.557
RACEHP2_P1	Other Single/Multiple Race vs White	1.486	1.059	2.085
SRSEX	Female vs Male	0.919	0.775	1.090
SRAGE_P1	Self-Reported Age	1.000	0.994	1.007
INTVLANG	Cantonese vs English	2.645	0.775	9.023
INTVLANG	Korean vs English	2.441	0.894	6.663
INTVLANG	Mandarin vs English	17.610	7.712	40.215
INTVLANG	Vietnamese vs English	88.361	29.383	265.715
INTVLANG	Spanish vs English	3.298	2.304	4.719
AD53_Difficulty	Yes vs No	2.328	1.803	3.006
AD57_Physical Limits	Yes vs No	2.052	1.507	2.796
Disabled	Yes vs No	1.609	1.241	2.085
DSTRS_P1	Serious Psychological Distress	1.131	1.105	1.157
Chronic Disease	Yes vs No	2.520	2.047	3.103
AK22_P	Income (sqrt)	0.997	0.996	0.998
ACMDNUM	Yearly Doctor visits	1.108	1.076	1.142
BMI_P	Body Mass Index	1.037	1.022	1.053

Odds Ratios for weighted logistic regression.

It is worth noting here, that in Fig. 3a and Table 1 we see evidence that the Fuzzy Forests model does not struggle with certain pathologies. For instance, note that we dropped the Tagalog respondents from these traditional analyses, because there were so few observations for them that logistic regression models were at best weakly-identified. We do not report those analyses here. However, including an indicator for taking the 2013 CHIS interview in Tagalog, with exactly the same logistic regression specification as reported in Fig. 3b produces an nonsensical estimate of 7.67, with a standard error of 1.54 (the other coefficients are similar to those reported in the text for the model without the Tagalog indicator). Worse still, we are unable to recover a valid odds ratio estimate for Tagalog versus English. Our Fuzzy Forests analysis, including the Tagalog respondents, produced results that are similar to those reported in the paper. Also note that for some of the language use parameters where we lack enough information (e.g. Cantonese and Mandarin speakers), the logistic regression analysis produces estimates with high variance.

Finally, we validated the model using the 2014 CHIS data as our test set. Note that the model was trained using only the 2013 data and we correctly predicted good health outcomes 86% of the time using the 2014 CHIS data. Similarly, when we ran the survey weighted logistic regression using the same model built from 2013 data and tested using the 2014 survey data (see Table 2 and Fig. 3b), the resulting area under the ROC curve 0.858. Both of these robustness checks suggest that the model trained on the 2013 data produces a good fit and suggesting that the model is generalizable.

**Table 2:** Odds Ratios and 95% Wald Confidence Limits for 2014 CHIS Data.

Parameter	Label	Point Estimate	95% Wald Confidence Limit	
RACEHP2_P1	African American vs White	1.245	0.858	1.808
RACEHP2_P1	American Indian/Alaskan vs White	1.678	0.679	4.148
RACEHP2_P1	Asian vs White	1.622	1.096	2.401
RACEHP2_P1	Latino/a vs White	1.594	1.135	2.239
RACEHP2_P1	Other Single/Multiple Race vs White	1.144	0.776	1.686
SRSEX	Female vs Male	0.827	0.669	1.023
SRAGE_P1	Self-Reported Age	1.003	0.996	1.01
INTVLANG	Cantonese vs English	8.396	3.958	17.810
INTVLANG	Korean vs English	3.041	1.282	7.213
INTVLANG	Mandarin vs English	3.262	1.060	10.036
INTVLANG	Vietnamese vs English	23.387	12.772	54.516
INTVLANG	Spanish vs English	3.351	2.370	4.738
AD53_Difficulty	Yes vs No	2.344	1.719	3.197
AD57_Physical Limits	Yes vs No	2.010	1.514	2.669
Disabled	Yes vs No	1.741	1.318	2.300
DSTRS_P1	Serious Psychological Distress	1.140	1.112	1.169
Chronic Disease	Yes vs No	2.467	1.967	3.095
AK22_P	Income (sqrt)	0.997	0.996	0.998
ACMDNUM	Yearly Doctor visits	1.101	1.069	1.133
BMI_P	Body Mass Index	1.064	1.045	1.082

Odds Ratios for weighted logistic regression using 2014 CHIS data.

## Discussion

Using Fuzzy Forests, we found response variations to outcome variables such as general health outcomes by interview language. These differences remained robust even after controlling for potential confounders such as age, gender, income and ethnicity. Our results, while focused on the associations between interview language and health outcomes, suggest that there may be cultural differences in the reporting of health outcomes, as documented in past research^15,16^. This should be studied further in subsequent research. Additionally, survey language needs to be examined and controlled for in other public health studies that offer respondents the opportunity to take a survey in different languages. Particularly in light of the fast-growing Latino population, researchers conducting surveys in both English and Spanish should be particularly aware of these cultural distinctions given that the Spanish speaking population in the United States is comprised of individuals hailing from more than ten different countries, each of whom have their own unique background and culture^24^. Even the process of translating an English survey into Spanish is no easy task — each Spanish speaker will have their own way of translating an item, and when concepts are especially complicated or prone to social desirability bias (such as health outcomes), the task becomes even more challenging. One potential solution, as suggested in the literature, is to use an anchoring vignettes approach^25,26^. In brief, this method asks survey respondents for a self-assessment of the concept being measured. Additionally, they would also be asked to assess several hypothetical individuals described by short vignettes on the same scale. Doing so could make it possible to create an interpersonally comparable measurement where the self-assessments could function as the “anchor”. One important caveat is that these vignettes should be structured such that the hypothesized ideal point should be unchanged when translated into different languages. This technique could then be applied to Asian languages.

As we discussed previously, analyzing the CHIS survey can be challenging given its complexity and size. Model selection in this case, using typical methods, can be time consuming and burdensome in that best subset selection may be intractable in a large data setting. Moreover, backwards and forwards selection yield differing sets of significant variables. In our analysis, the more traditional survey weighted logistic yielded different results with forwards and backwards selection. Both methods gave a large number of potential predictors, many of which were tied as they were given *p* values < 0.001. This could make it difficult for a researcher to choose among the potential predictors. We have demonstrated that Fuzzy Forests offers a relatively fast and computationally feasible alternative to weighted logistic variable selection and screening. This approach also shows the value in novel machine learning methods in complex survey settings in that it shows the network of relatedness of the potential predictors. Fuzzy Forests also has value over Random Forests in that it is known that Random Forests variable selection is biased under correlation^27–29^. If the correlation is unknown, Fuzzy Forests can yield relatively unbiased variable importance while yielding insights into the data structure through it correlation network. Knowing which variables cluster together and which of those are important in prediction can yield insights into the mechanisms behind the response and could potentially help drive future surveys. The predictive accuracy in using the model trained on 2013 data to predict 2014 outcomes when the percentage of non-English interviews increased dramatically also help strengthen the argument that the model is generalizable and there indeed may be a cultural effect in interview language questions.

Using a machine learning approach like the Fuzzy Forests is useful when a researcher suspects that survey responses to important substantive questions may be quite different due to issues of survey implementation and there is concern that there may be correlation among the predictors. To the best of our knowledge, the work reported here is the first to use a machine learning approach like the Fuzzy Forests to find and analyze survey response heterogeneity that results from survey design issues. While other “fuzzy” estimation approaches have been used to study uncertainty in survey responses, those approaches are studying a different and quite specific problem, and use very different methodologies than the Fuzzy Forests. For example, Wagner *et al*. use a “fuzzy sets” approach to examine interval-response data from surveys^30^. Such survey response options are sometimes implemented when researchers wish to study respondent uncertainty or ambivalence, which is distinct from the the type of response heterogeneity we are studying in the CHIS data based on language-of-interview. For further elaboration of uncertainty in survey responses and interval-response data, see^9^ and^31^. We believe that machine learning tools like the Fuzzy Forests, which combine both unsupervised and supervised learning techniques, offer potential for the screening of important variable and the analysis of survey design and implementation effects on survey responses; for example, survey mode effects, or race and gender of interviewer effects.

Furthermore, our work has implications for survey design, in particular, for the process of developing and testing survey instruments. For example, a researcher who wanted to implement a survey in multiple languages could pilot test the instrument, and use Fuzzy Forests to test for survey response heterogeneities based on language-of-interview. The researcher could then examine the translation process that produced the multilingual questionnaire, audit the training of the interviewers and their language skills, or undertake other analyses to diagnose the source of the linguistic differences in survey response. Alternatively, those who implement large-scale survey projects (like the CHIS) could use Fuzzy Forests to find language-based heterogeneities, and use the results from those analyses to improve future implementations of these important surveys. Our approach could be used to achieve the type of “conceptual equivalence” (where the translated measures are shown to measure the same underlying theoretical concepts) which was the goal when the CHIS was originally translated into multiple languages^32,33^.

Fuzzy Forests is a novel machine learning algorithm that can automatically and systematically be used for screening important variables in complex surveys. This technique can provide insights that may not emerge when using more conventional methods. Fuzzy Forests are non-linear and nonparametric. Their construction takes into account interactions in a limited fashion, and eliminates the need for specifying them a priori. The final recursive feature elimination step allows for interactions between modules to be accounted for in the model. Of course, there are several caveats to this process. Random Forests are composed of regression trees, greedy algorithms that are not guaranteed to find a global optimum. Numerous tuning parameters exist in Fuzzy Forests, especially in the module formation. While our results appear to be robust to changes in the module structure, that is not guaranteed in every application. Supplemental Figs 4 and 5 gives the variable importance when using a random assignment to the modules as well as the variable importance plot from Random Forests respectively. Note than in all of these analyses, INTV (interview language) is among the list of important variables.

Researchers must also ensure that they are building enough trees in their forest, especially when there are many parameters. One needs to set the number of trees to ensure, with reasonable certainty, that with the *mtry* selected, each parameter has a chance of entering the model. One must also realize that the relative ranking must be taken with a grain of salt. These techniques help elucidate which set of variables are important in terms of prediction and also aid in interpretation via the correlation networks. However caution is warranted when using any machine learning algorithm, one must ensure that the scientific merit is there and not perform these analysis without a reasonable degree of caution for its generalization and due consideration to the tuning parameters.

Our analysis also shows that the application of Fuzzy Forests in the CHIS data uncovered important differences in public health survey outcome variables, associated with the language used by the interview respondents. Substantively, as an increasing number of surveys and polls are allowing respondents to use languages other than English to complete the survey, our findings show that researchers using those datasets should be aware of language-of-interview differences in their survey and try to control for these differences in their analyses. Depending on the nature of the language-of-interview differences, researchers might allow for heterogeneities through interaction terms or by using independent estimation models for respondents using different interview languages. Additionally, our work adds to a growing body of research on language-of-interview effects in surveys; clearly, much more work needs to be done to better understand why language-of-interview effects occur, and when they are most likely to cause heterogeneities in survey responses. As survey and poll designs grow ever more complex, for example using combinations of contact and interview modes, tools like the Fuzzy Forest could to be used by applied researchers to help detect methodological and survey contextual variables and aid in understanding how they are associated with survey responses.

## Methods

### CHIS

We use data from the publicly available California Health Interview Study (CHIS), a random-digit dial telephone survey (with about 20% of the subjects interviewed on mobile phones, 80% on landlines) that provides representative data on the state’s non-institutionalized population in all 58 counties in California. Details and publicly available data from the CHIS are available online, http://healthpolicy.ucla.edu/chis. The CHIS uses a multi-stage sampling design, so that it can achieve geographic coverage across the entire state and across many subpopulations (especially racial and ethnic minority groups in California). It is the largest health interview survey in the US and provides rich data on the health needs and health care usage of California residents. Given the fact that the CHIS contains detailed micro-data on public health, it is used in a large array of research studies, including smoking^34^, epilepsy^35^, domestic violence among sexual minorities^36^, childhood asthma^37^, hereditary cancer^38^, and many other public health issues. Specifically, we use the publicly available data from the 2013 and 2014 CHIS Adult Surveys, which we treat as independent surveys; where the 2013 data is used for training and the 2014 data is used for testing. The 2013–2014 CHIS survey was fielded beginning in February 2013, running through the beginning of January 2013, with about half of the subjects interviewed in 2013 and the other half interviewed in 2014.

One of CHIS’s goals is to achieve widespread coverage of California’s ethnically and racially diverse population. In fact, ethnic and racial minorities now comprise the majority of state’s population– Latinos are the largest at 38.8%, followed by Asian at 14.7% and Blacks at 6.5%. The CHIS utilizes different approaches in its sampling scheme to try to ensure widespread coverage of racial and ethnic minority groups, in particular by offering the survey in a multitude of languages, other than English: Spanish, Vietnamese, Korean, Cantonese, Mandarin, and Filipino/Tagalog. That the CHIS allows respondents to complete the interview in so many different non-English languages makes it an excellent laboratory for the study of language effects in large surveys. Table 3 provides summary statistics on language-of-interview in the 2013 and 2014 CHIS. In the 2013 data, the sample included 20,724 respondents: 90.18% of those respondents completed the interview in English. That same year, 8.24% of the interviews were completed in Spanish (1,707 interviews), with 328 interviews completed in an Asian language (1.58%). The 2014 data had slightly fewer total interviews (19,516), and fewer English-language interviews (87.27%). There were 1,619 Spanish interviews in the 2014 data (8.30%), and 865 Asian interviews (an increase in Asian interviews to 4.43%). While we would have preferred a larger number of non-English interviews in order to study variability in survey responses, the 2013–2014 CHIS data contains an ample number of non-English responses for our analysis.

**Table 3:** Language of Interview in 2013–2014 CHIS data.

Language	2013 Interviews		2014 Interviews	
	N	Percent	N	Percent
English	18,689	90.18	17,032	87.27
Spanish	1,707	8.24	1,619	8.30
Vietnamese	54	0.26	348	1.78
Korean	147	0.71	156	0.80
Cantonese	68	0.33	115	0.59
Mandarin	58	0.28	219	1.12
Filipino/Tagalog	1	0.00	27	0.14
Total	20,724	100.00	19,516	100.00

Data from 2013–2014 CHIS surveys.

The CHIS data are observational, and thus we must rely on the available features in the public-release datasets. To ensure that our analysis is estimating language differences in health outcome responses in these survey data, and not demographic population differences, we use a wide array of demographic and behavioral features from the CHIS. By including population and other health behavior features in the Fuzzy Forests model (as we describe below), we increase our confidence in our model’s ability to estimate language differences in the health outcomes that we analyze.

The data are de-identified and publicly available for download. The use of these data has been approved by the UCLA Institutional Review Board (IRB 18-001941), and by the Caltech Institutional Review Board (IRB 18-0849). All of the terms of use of these data have been met.

We use the *fuzzyforest* package in R to estimate the Fuzzy Forests results reported in the paper. We use *SAS Version 9.3 Proc Surveylogistic* to estimate the weighted logistic regression results reported here.

### Fuzzy Forests

As data becomes easier to generate, many fields are faced with an overabundance of information^39^. In many cases there may be many more parameters than observations, and in a majority of applications these parameters are correlated (sometimes strongly correlated). With the recent development of sophisticated variable selection models, researchers now often use these methods to see which variables are important in predicting their outcome of interest. For a thorough review of variable selection models, see Chapter 3 in^40^. Variable selection has been used in many diverse fields to select features that increase predictive accuracy while reducing noise. One popular method of variable selection is the LASSO (least absolute shrinkage and selection operator). LASSO imposes a constraint, *λ*, on the size of the regression coefficients *β* in ordinary least squares (OLS regression). That is, LASSO imposes a constraint on the absolute value of the sum of the *β*’s, the higher the value of *λ* the more the coefficients are shrunk towards zero. The value of *λ* is often determined through cross-validation. In that way, LASSO is performing variable selection. Ridge regression is similar but it does not force the *β*’s to be zero. Under correlation, LASSO tends to randomly pick one from a set of highly correlated variables and sets the others towards zero. Further, the coefficients may not be unique under correlation. This may be undesirable when you want to produce a stable list of variables that are driving the signal when some of those variables might be correlated^41,42^. These models also require the user to specify the model a priori including any possible interactions.

Random Forests (RF)^43^, a popular machine learning technique, can handle situations where the number of parameters greatly exceed the number of observations. It is also non-linear and non-parametric. Variable selection is not an integral part of the model development but is rather a consequence of the model building procedure. Importance is often assessed by examining the mean decrease in predictive accuracy when a variable is randomly permuted, thus distorting its signal to the outcome variable, and compared to its original form leaving all other variables unchanged. The idea is that if a variable is truly important to prediction, then the average accuracy should be diminished when that variables’ relationship with the outcome is destroyed. It is important to note that the accuracy is determined using the out-of-bag samples and averaged across all bootstrap samples. Further adding to its popularity, RF predictive accuracy often beats LASSO’s, especially when the features are correlated and the relationships among them non-linear. However, Random Forest (RF) variable selection is known to be biased when there is high correlation among the covariates^27^. When a dataset contains both correlated and independent variables, the RF variable importance tends to rank the correlated variables higher than the independent variables; when there are a large number of variables, the independent variables are often assessed with little to no variable importance when there are a large number of covariates.

Fuzzy Forests is a novel machine learning algorithm that extends Random Forests and was specifically designed for feature (variable) selection when the features are highly correlated^29,44^. Fuzzy Forests are thus well-suited for high dimensional problems, where multicollinearity is an issue. Furthermore, Fuzzy Forests do not assume normality, and can be used where features or variables have interactive or nonlinear relationships with respect to the outcome of interest.

Fuzzy Forests works in two steps: first, a screening step where the features are clustered into modules by similarities in their correlation structure such that features within each module are highly correlated and the modules are roughly independent from each other; second, a recursive feature elimination random forest selection step where the important features are selected from each module. Then, all the surviving features are placed in a final recursive feature elimination step. Supplemental Fig. 2 gives a graphical representation of Fuzzy Forests. Fuzzy Forests allows for two implementations of correlation or network structure: (1) Weighted Correlation Network Analysis (WGCNA), which allows the data to specify the modules (using the *wff* call); or (2) the analyst can specify the correlation modules themselves, if they have an a priori reason to justify the correlation modules (using the *ff* call). The latter function may be useful in surveys, like CHIS, that cluster questions of similar content by section.

CHIS contains a large number of features with possibly unknown interactions between these potentially correlated covariates and the outcome variables. As module formation is itself a tuning parameter, with WGCNA especially, we tried many different module structures (including random assignment and networks), and found similar end results for each approach. Ultimately we formed modules with a power of 7 to form approximate scale-free topology. We merged any module with more than 50 percent similarity. Supplemental Tables 1–9 list the variables that were included in each module, while Supplemental Table 10 lists the different sections of the CHIS surveys. Within each module, we used recursive feature selection to determine the top thirty five percent best predictor covariates in each module. The survivors from each module were then placed into a single recursive feature elimination forest and the top twenty predictors selected. We also tried numerous values for the number of variables for splitting at tree nodes (the *mtry* parameter), and found similar results for the different values we specified.

In this application, we used the outcome variable “General Health Outcome”, defined on a five-point scale: Excellent, Very Good, Good, Fair, and Poor. In the survey, this is survey item AB1. We then created a binary variable for good health outcomes which was coded as 1 if the person responded “Excellent, Very Good or Good” and 0 otherwise. We included all variables as potential predictors. Note that we drop respondents who took the CHIS in Tagalog, as in our validation analysis using logistic regression their inclusion leads to weakly-identified parameters.

Using the module structure created by constructing a weighted correlation network, we found the top twenty parameters for “good health outcomes” by Fuzzy Forests. We chose different random seeds and repeated the process 1,000 times. For validation, we put the model into a traditional logistic regression with the survey weights and adjusted for known confounding variables such as age, sex, bad health outcomes (a binary indicator that equals 1 if a patient has been diagnosed with, taken medication for, or ever visited the Emergency Room for cardiovascular disease, high blood pressure or (inclusive or) diabetes), difficulties in everyday life feeding/dressing or mentally, income and ethnicity. For further validation, we trained a Fuzzy Forests with the 2013 CHIS data only, and tested the model using the 2014 CHIS data as the test set.

## Supplementary information

Supplementary Information

## Data Availability

The data are available at the CHIS website http://healthpolicy.ucla.edu/chis/data/Pages/GetCHISData.aspx. The code required for replicating the results reported in this paper is available at: https://github.com/OHDSI/FuzzyForest.
