# Supplementary material for: Using Machine Learning to Uncover Hidden Heterogeneities in Survey Data
Source: Sci Rep. 2019 Nov 5;9:16061. doi: 10.1038/s41598-019-51862-x (PMC6831673; doi:10.1038/s41598-019-51862-x)
Supplement: Supplementary file 1 — Supplementary Information [file 41598_2019_51862_MOESM1_ESM.pdf]

Using Machine Learning to Uncover Hidden Heterogeneities in Survey Data: Supplementary Information

Christina M. Ramirez[1,*+], Marisa A. Abrajano[2+], and R. Michael Alvarez[3+]

[1]Department of Biostatistics, UCLA School of Public Health, UCLA, Los Angeles, CA 90095-1772
[2]Department of Political Science, University of California, San Diego, La Jolla, CA 92093-0521
[3]Division of Humanities and Social Sciences, California Institute of Technology, Pasadena, CA 91125
[*]corresponding author cr@g.ucla.edu

ABSTRACT

Survey responses in public health surveys are heterogeneous. The quality of a respondent's answers depends on many factors, including cognitive abilities, interview context, and whether the interview is in person or self-administered. A largely unexplored issue is how the language used for public health survey interviews affects the survey response. We introduce a machine learning approach, Fuzzy Forests, which we argue is well-suited for estimation of heterogeneity in survey responses. We use the 2013 California Health Interview Survey (CHIS) as our training sample and the 2014 CHIS as the test sample. We found that non-English language survey responses differ substantially from English responses in reported health outcomes. We also found heterogeneity among the Asian languages suggesting caution should be used when interpreting results when these languages are grouped together. The 2013 fuzzy forest model correctly predicted 86% of good health outcomes using 2014 data. We show that the Fuzzy Forest methodology is potentially useful for eliciting and understanding other types of survey response heterogeneity. This is especially true in high-dimensional and complex surveys.

Supplementary Information

Supplementary Figure 1 shows the variable importance results from Fuzzy Forests. Variables are ranked from the most to least important.

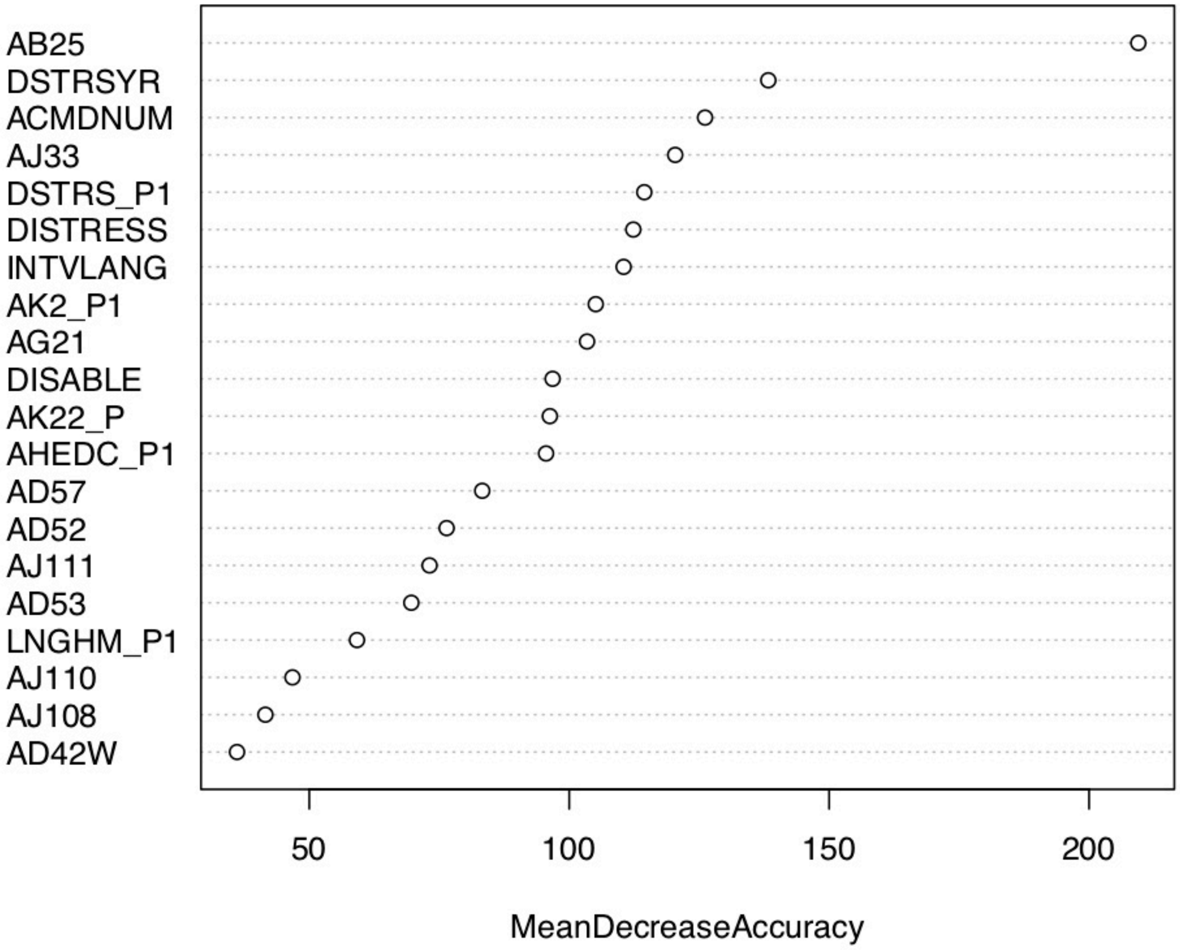

Supplementary Figure 1: Fuzzy Forest Results sorted by importance ranking from greatest to least.

Supplementary Figure 2 shows the dendogram created from the hierarchical clustering of the module eigenfactors. Module eigenfactors are the first principle component of each module matrix. Modules with similarities of 50 percent or more were merged.

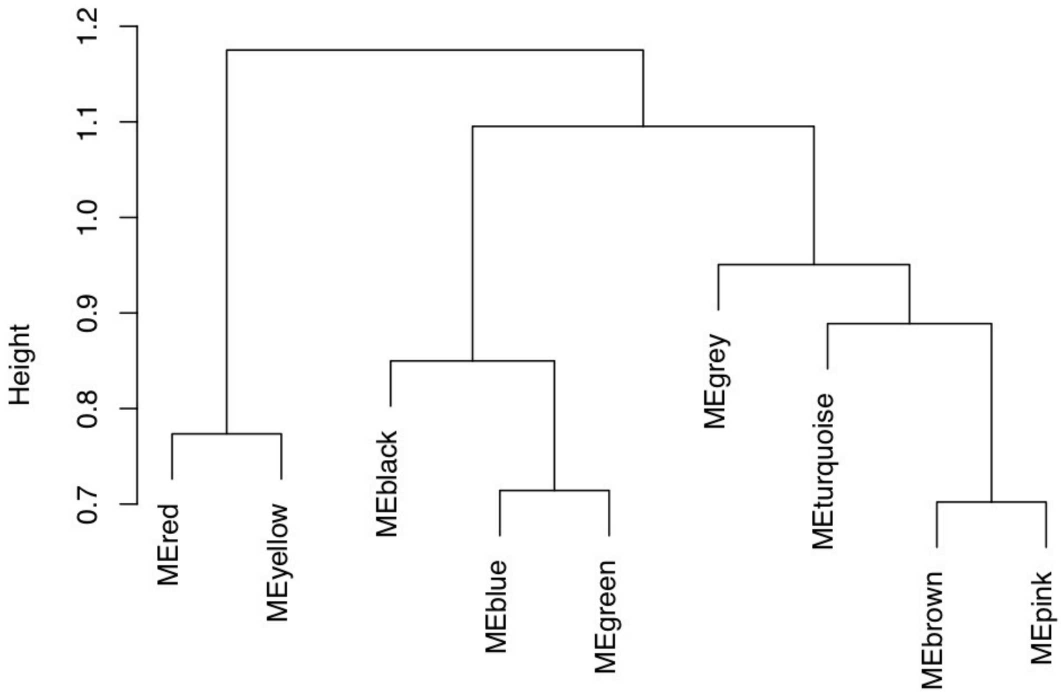

Supplementary Figure 2: Hierarchical Clustering of the Modules

Supplementary Figure 3 shows the ModPlot from Fuzzy Forests. This illustrates the percentage of "important variables" selected from each module from the weighted correlation network created by Fuzzy Forest

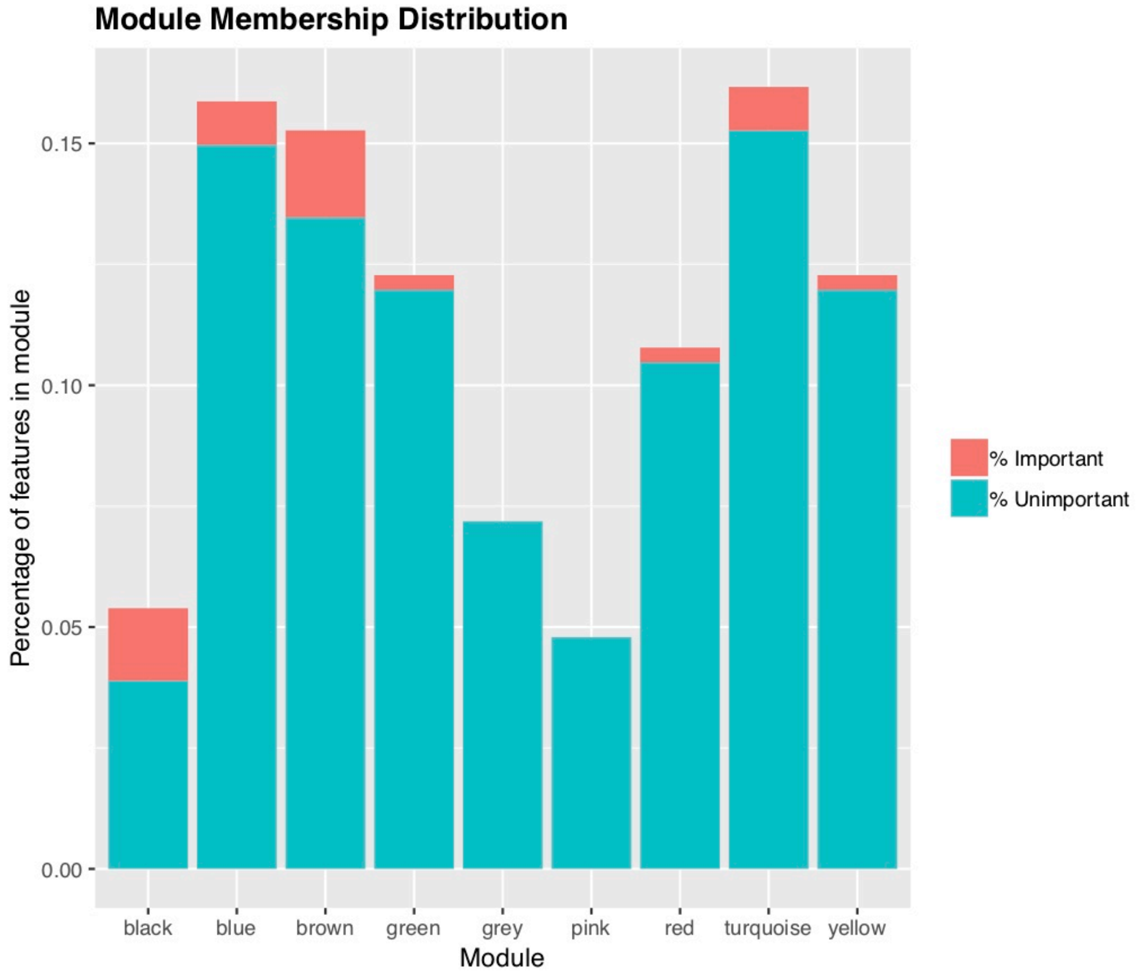

Supplementary Figure 3: ModPlot from Fuzzy Forest

Supplementary Figure 4 shows Fuzzy Forests Variable Importance Ranking when using random module assignment. Note that interview language (INTVLANG) is still among the list of important variables.

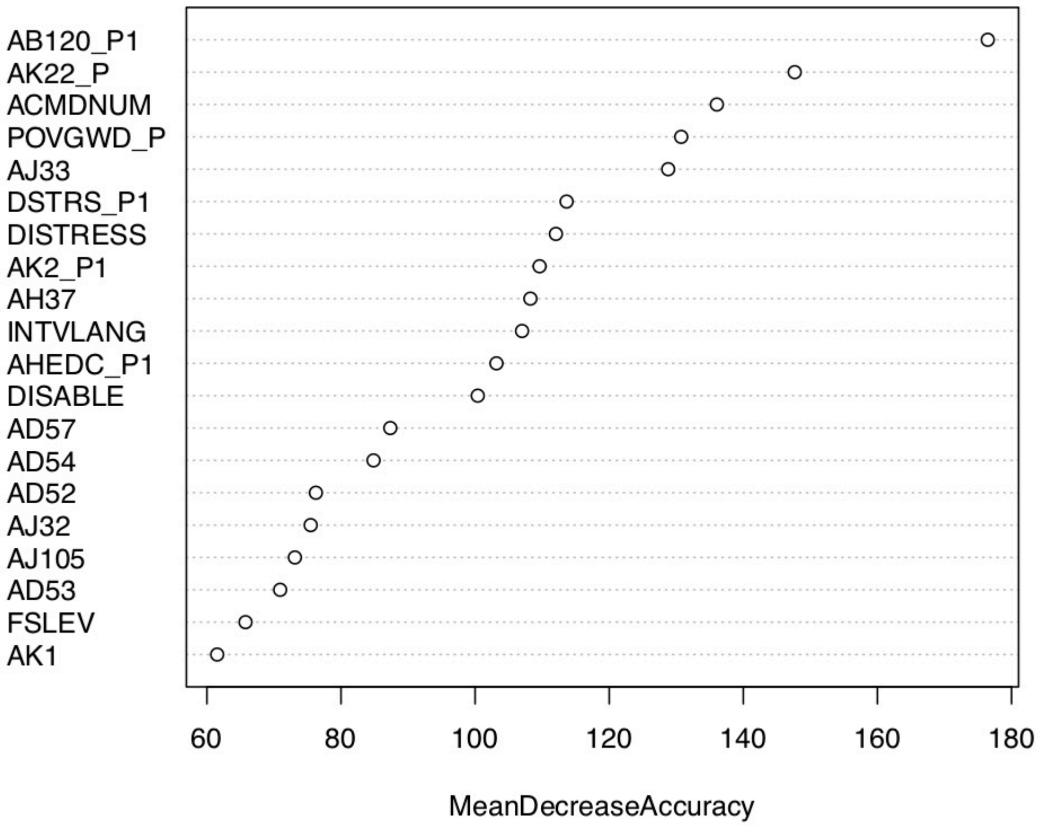

Supplementary Figure 4: Variable Importance Plot from Fuzzy Forest using Random Module Assignment

Supplementary Figure 5 shows the Random Forest Variable Importance plot. Note that interview language (INTVLANG) is among the list of important variables.

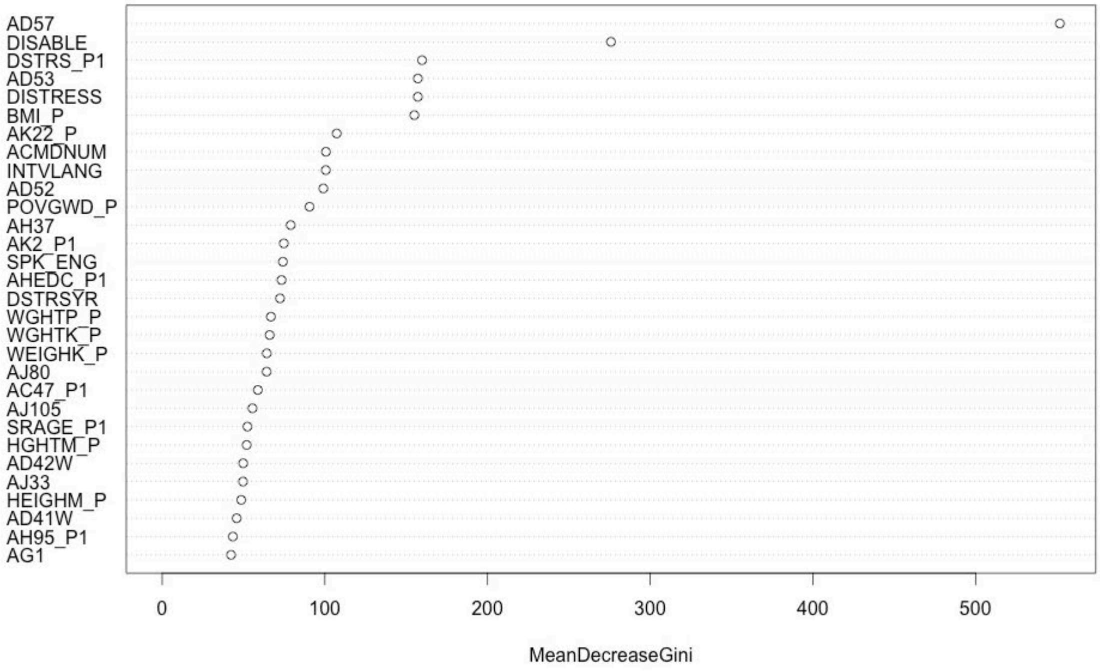

Supplementary Figure 5: Random Forest Variable Importance Plot

Supplementary Figure 6 shows the schematic for the work flow of Fuzzy Forests. Note that if users choose the *wff* call, then the modules are formed using correlation networks. The *ff* call allows the user to pre-specify the modules directly.

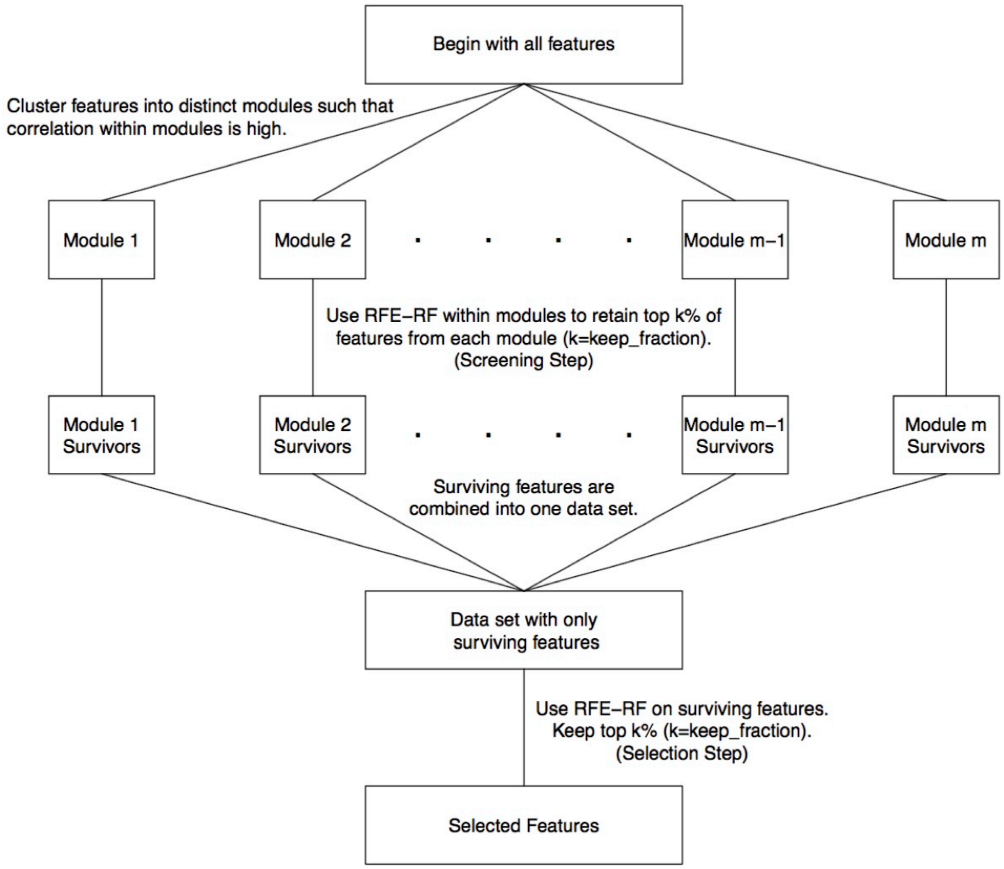

Supplementary Figure 6: Flow Chart of Fuzzy Forest

Supplementary Table 1: “Black” Fuzzy Forest Module

CHIS Feature	CHIS Feature Label
AD51	HAS DIFFICULTY LEARNING, REMEMBERING, CONCENTRATING
AD52	HAS DIFFICULTY DRESSING, BATHING, GETTING AROUND
AD53	HAS DIFFICULTY GOING OUTSIDE HOME ALONE
AD57	CONDITION LIMITS BASIC PHYS ACTIVITY
AF62	ANY MONTH PAST 12 MONTHS FELT WORSE
AH12	VISITED EMERGENCY ROOM FOR OWN HEALTH PAST 12 MOS
AH14	PATIENT IN HOSP OVERNIGHT DURING PAST 12 MOS
AJ29	FEEL NERVOUS PAST 30 DAYS
AJ30	FEEL HOPELESS PAST 30 DAYS
AJ31	FELL RESTLESS PAST 30 DAYS
AJ32	FEEL DEPRESSED PAST 30 DAYS
AJ33	HAD TROUBLE FINDING GENERAL DOC PAST 12 MOS
AJ34	FEEL WORTHLESS PAST 30 DAYS
DISABLE	DISABILITY
DSTRS12	LIKELY HAS HAD PSYCHOLOGICAL DISTRESS IN THE LAST YEAR
DSTRS30	LIKELY HAS HAD PSYCHOLOGICAL DISTRESS IN THE PAST MONT
ER	ER VISIT WITHIN THE PAST YEAR
PROXY	A PROXY INTERVIEW

Black module from the weighted correlation network estimated during the first stage of Fuzzy Forests using data from 2013-2014 CHIS surveys. This module encompasses variables dealing with difficulties with everyday activities, likely psychological distress and mental health issues.

Supplementary Table 2: “Blue” Fuzzy Forest Module

CHIS Feature	CHIS Feature Label
AC42	How often can you find fresh fruits and vegetables in your neighborhood?
AC42_P	HOW OFTEN FIND FRESH FRUIT-VEG IN (Recode)
AC44	How often are the fresh fruits and vegetables you find in your neighborhood affordable?
ACHLDS_P1	AMOUNT SPENT ON CHILD CARE PER WEEK (PUF
AD41W	In the past 7 days, how many times did you do that? Walk for at least 10 minutes for fun, relaxation, exercise, or to walk the dog
AD42W	How long did that walk take/On average, how long did those walks take
AE15	SMOKES CIGARETTES EVERYDAY, SOME DAYS OR NOT AT ALL
AG11	Does your spouse/partner usually work
AG8	Employment spouse/partner: Which of the following was your spouse/partner doing last week
AG9_P1	TYPE OF EMPLOYER ON SPOUSE’S MAIN JOB
AH16	During the past 12 months, did you delay or not get a medicine that a doctor prescribed for you?
AH22	During the past 12 months, did you delay or not get any other medical care you felt you needed— such as seeing a doctor, a specialist, or other health professional?
AH44	Is your spouse/partner also living in your household?
AH44A	In the past month, did you use any paid childcare for any children under age 14 while you or your spouse/you or your partner/you worked, were in school, or looked for work?
AHEDC_P1	EDUCATIONAL ATTAINMENT (PUF 1 YR RECODE)
AI37A	You said that you have a Medicare Supplement plan. Does your partner/husband/wife/spouse also have a Medicare supplemental policy?
AI45_P1	MAIN REASON SPOUSE NOT IN EMP HEALTH PLAN (PUF 1 YR RECODE)
AI45A_P1	MAIN REASON SPOUSE INELIGIBLE EMP HEALTH PLAN (PUF 1 YR RECODE)
AJ133	HAD TROUBLE FINDING GENERAL DOC PAST 12 MOS
AK10A_P	SPOUSE’S EARNINGS LAST MONTH (PUF RECODE)
AK22_P	HOUSEHOLD’S TOTAL ANNUAL INC (PUF RECODE)
AK33_P1	# OF PERSONS SUPPORTED BY HH INCOME NOT IN HH (PUF 1 YR
AM20	People in this neighborhood generally do NOT get along with each other.
SRAI	SELF-REPORTED AMERICAN INDIAN
ASTCUR	CURRENT ASTHMA STATUS
FAMSIZE2_P1	FAMILY SIZE: INCL. ALL PEOPLE SUPPORTED by HH income
FAMTYP_P	FAMILY TYPE (PUF RECODE)
FORGO	HAD TO FORGO NECESSARY CARE
FPG	FEDERAL POVERTY GUIDELINE
HEIGHM_P	HEIGHT: METERS - UCLA (PUF RECODE)
HGHTI_P	HEIGHT: INCHES (PUF RECODE)
HGHTM_P	HEIGHT: METERS (PUF RECODE)
HHSIZE_P1	HOUSEHOLD SIZE (PUF 1 YR RECODE)
INS_S	SPOUSE CURRENTLY INSURED
INS64S_P	SPOUSE’S CURRENT HEALTH COVG - < 65 Y
INS65_S	Type of insurance spouse 65+
INSMD	COVERED BY MEDI-CAL
INSOG	COVERED BY OTHER GOVT PLANS
INSPS_2	PRIMARY OR SECONDARY COVERAGE
OFFTK_S	OFFER, ELIGIBILITY, ACCEPTANCE OF employer based ins
POVGWD_P	FAMILY POVERTY THRESHOLD LEVEL (PUF RECODE)
POVLL	Poverty Level
POVLL_ACA	FAMILY POVERTY THRESHOLD LEVEL: ACA MAGI ELIGIBILITY (4 LVLS)
SMKCUR	CURRENT SMOKER
SMOKING	CURRENT SMOKING HABITS
SRAA	Self reported- African American
UR_BG	RURAL AND URBAN - CLARITAS (BY BLOCK GROUP)
UR_TRACT	RURAL AND URBAN - CLARITAS (BY CENSUS TRACT)

Blue module from the weighted correlation network estimated during the first stage of Fuzzy Forests using data from 2013-2014 CHIS surveys. This module encompasses variables dealing with neighborhood characteristics, delaying health care, governmental or employer based health care coverage, smoking status, and household members.

Supplementary Table 3: “Brown” Fuzzy Forest Module

CHIS Feature	CHIS Feature Label
AB81	DOCTOR TOLD HAD DIABETES ONLY DURING PREGNANCY
AC32	HAD ALCOHOL PAST 12 MONTH
AD40W	WALKED AT LEAST 10 MIN FOR LEISURE PAST 7 DAYS
AG1	TIME SINCE LAST VISIT TO DENTIST/SPECIALIST
AG10	RESPONDENT USUALLY WORKS
AG21	LANGUAGE OF TV, RADIO, NEWSPAPERS
AG22	EVER SERVE IN U.S. ARMED FORCES
AG3	HAVE ANY KIND OF DENTAL INSURANCE
AH1	HAVE USUAL SOURCE OF HEALTH CARE
AH37	LEVEL OF ENGLISH PROFICIENCY: GENERAL
AJ10	NEEDED SOMEONE ELSE TO HELP UNDERSTAND DOCTOR
AJ105	KNOW RIGHTS TO INTERPRETOR DURING MED VISIT
AJ108	EVER USED INTERNET
AJ11	WHO HELPED YOU UNDERSTAND DOCTOR
AJ111	SOMEONE TO HELP FILL OUT APPL ONLINE
AK23	LIVE IN HOUSE, DUPLEX, BUILDING WITH 3+ UNITS OR MOBILE HOME
AK25	OWN OR RENT HOME
AK32	HH INCOME SUPPORTS SOMEONE LIVING IN U.S. BUT NOT IN HH
AL18A	RECVD SOCIAL SECURITY OR PENSION LAST MONTH
AL2	RECEIVING TANF OR CALWORKS
AL32	RECD ANY WORKERS COMPENSATION LAST MO
AL5	RECEIVING FOOD STAMP BENEFITS
AL6	RECEIVING SSI (SUPPLEMENTAL SECURITY INCO
AL7	Are you on WIC?
AM1	HOW OFTEN FOOD DIDN’T LAST,COULDN’T AFFORD MORE, PAST 12 MOS
AM3	ALL ADULTS CUT/SKIPPED MEALS IN PAST 12 MOS FOR MONEY
AM36	DID VOLUNTEER WORK OR COMMUNITY SERVICES PAST YR
AM39	VOLUNTEER IN ORG DEALING W/COMM PROB PAST YR
AM3A	HOW OFTEN ALL ADULTS CUT/SKIPPED MEALS IN PAST 12 MOS FOR MONEY
AM4	ATE LESS THAN SHOULD BECAUSE OF MONEY IN PAST 12 MOS
AM40	MEET INFORMALLY TO DEAL W/COMM PROB PAST YR
AM5	HOW OFTEN HUNGRY BUT DIDN’T EAT B/C OF MONEY IN PAST 12 MOS
SRSEX	SELF-REPORTED GENDER
SRTENR	SELF-REPORTED HOUSEHOLD TENURE
SRW	SELF-REPORTED WHITE
MARIT	MARITAL STATUS- 3 CATEGORIES
INSEM	COVERED BY EMPLOYER BASED PLANS
AH33NEW	BORN IN U.S.
CITIZEN2	CITIZENSHIP STATUS (3 LVLS)
MARIT2	MARITAL STATUS- 4 CATEGORIES
SPK_ENG	ENGLISH USE AND PROFICIENCY
FSLEV	FOOD SECURITY STATUS LEVEL
FSLEVCB	FOOD SECURITY STATUS (2 LVLS)
INTVLANG	LANGUAGE OF INTERVIEW
LATIN2TP	LATIN/HISPANIC SUBTYPES - 2 LVLS
AJ50_P	LANGUAGE DOCTOR SPEAKS TO R
AK2_P1	MAIN REASON FOR NOT WORKING LAST WEEK
AJ142_P1	MAIN TYPE OF BIRTH CONTROL RECEIVED FROM DR PAST YR
LNGHM_P1	LANGUAGE SPOKEN AT HOME
YRUS_P1	YEARS LIVED IN THE U.S

Brown module from the weighted correlation network estimated during the first stage of Fuzzy Forests using data from 2013-2014 CHIS surveys. This module encompasses variables dealing with food insecurity, SSI and food stamp benefits, citizenship status and length in the US and language questions including interview language.

Supplementary Table 4: “Green” Fuzzy Forest Module

CHIS Feature	CHIS Feature Label
AB22	DOCTOR EVER TOLD HAVE DIABETES
AB29	DOCTOR EVER TOLD HAVE HIGH BLOOD PRESSURE
AB34	DOCTOR EVER TOLD ANY HEART DISEASE
AB99	DOC EVER TOLD HAVE PRE- OR BORDERLINE DIABETES
AC34_P1	# DAYS HAD 5+ DRINKS PAST 12 MONTHS
AC35_P1	# DAYS HAD 4+ DRINKS PAST 12 MONTHS
AD50	BLIND/DEAF OR HAS SEVERE VISION/HEARING PROBLEM
AD54	HAS DIFFICULTY WORKING AT A JOB
AH71_P1	HEALTH PLAN DEDUCTIBLE MORE THAN $1,000
AH72_P1	HEALTH PLAN DEDUCTIBLE MORE THAN $2,000
AH73B	SPECIAL FUND TO PAY MEDICAL EXPENSES
AH74	TRIED TO FIND HEALTH INSURANCE ON OWN
AH96_P1	HEALTH PLAN DEDUCTIBLE > $2,000
AH97_P1	PLAN DEDUCTIBLE COVERING ALL PERSONS >$4,000
AI15	MAIN REASON NOT IN EMPLOYER’S HEALTH PLAN
AI15A	MAIN REASON INELIGIBLE FOR EMPLOYER’S HEALTH PLAN
AI33_6	Was your other health insurance Medi-CAL, Healthy Families, a plan you obtained through an employer, a plan you purchased directly from an insurance company, a plan you purchased through Covered California, or some other plan?
AJ110	CONFIDENCE TO COMPLETE APPL ONLINE ON OWN
AJ140	RECEIVED BIRTH CONTROL INFO FROM DR PAST YR
AJ143_01	PLACE RECEIVED MAIN BIRTH CONTROL METHOD/RX
AJ144	RECEIVED BIRTH CONTROL INFO FROM DR PAST YR
AJ145	RECEIVED MALE BIRTH CONTROL METHOD FROM DOC PAST YR
AK10_P	SPOUSE’S EARNINGS LAST MONTH
AK4	TYPE OF EMPLOYER AT MAIN JOB
AK7_P1	LENGTH OF TIME WORKING AT MAIN JOB
AK8	# PEOPLE WORKING FOR EMPLOYER/YOU
AKWKLNG	TIME AT MAIN JOB
BINGE12	BINGE DRINKING IN PAST YEAR.
IND_FLAG	
INDMAIN2	
INS64_P	HEALTH COVERAGE - < 65
INSLT_P	HEALTH INS COVG LAST 12 MOS, W/ CURRENT STATUS
INSMC	COVERED BY MEDICARE
INSPS	PRIMARY OR SECONDARY COVERAGE
INST_12	HEALTH INS COVERAGE IN LAST 12 MOS,
INSTYP_P	TYPE OF CURRENT HEALTH COVERAGE SOURCE FOR ALL AGES
OCC_FLAG	
OCCMAIN2	
OFFTK	OFFER, ELIGIBILITY, ACCEPTANCE OF employer based ins
UNINSANY	UNINSURED IN PAST 12 MOS

Green module from the weighted correlation network estimated during the first stage of Fuzzy Forests using data from 2013-2014 CHIS surveys. This module encompasses variables dealing with chronic health conditions, binge drinking, birth control, employment and health insurance coverage.

Supplementary Table 5: “Grey” Fuzzy Forest Module

CHIS Feature	CHIS Feature Label
AB17	DOCTOR EVER TOLD HAVE ASTHMA
AH100	ANYONE HELP FIND HEALTH PLAN
AJ134	NOT ACCEPTED AS NEW PATIENT BY DOC PAST YR
AJ146	PLACE RECEIVED MAIN BIRTH CONTROL METHOD
SRAS	SELF-REPORTED ASIAN
SRCH	SELF-REPORTED CHINESE
SRKR	SELF-REPORTED KOREAN
SRPH	SELF-REPORTED FILIPINO
SRVT	SELF-REPORTED VIETNAMESE
SRASO	SELF-REPORTED OTHER ASIAN GROUP
UR_CLRT	RURAL AND URBAN - CLARITAS (BY ZIPCODE)
UR_CLRT2	RURAL AND URBAN - CLARITAS (BY ZIPCODE)
UR_IHS	RURAL AND URBAN - IHS
UR_OMB	RURAL AND URBAN - OMB
UR_RHP	RURAL AND URBAN - RHP
“IHS"	COVERED BY INDIAN HEALTH SERVICES
PC_NEWP	PRIMARY CARE: HAVE DIFFICULTY FINDING A PROVIDER THAT NEW PATIENTS
OVRWT	OVERWEIGHT OR OBESE
AH101_P	PERSON WHO HELPED FIND HEALTH PLAN
AC48_P1	# OF GLASSES OF NON-LOW/FAT MILK DRANK YESTERDAY
AC47_P1	# OF GLASSES OF WATER DRANK YESTERDAY
AH98_P1	DIFFICULTY FINDING PLAN WITH NEEDED COVERAGE
AH99_P1	DIFFICULTY FINDING PLAN THAT IS AFFORDABLE
SRJP	SELF-REPORTED JAPANESE

Grey module from the weighted correlation network estimated during the first stage of Fuzzy Forests using data from 2013-2014 CHIS surveys. The Grey module, by construction includes variables that did not have high similarity with other variables.

Supplementary Table 6: “Pink” Fuzzy Forest Module

CHIS Feature	CHIS Feature Label
AH103	BUY HEALTH PLAN DIRECTLY FROM INS CO/HMO
AH43A	LIVING WITH A PARENT
AH6	HOW LONG SINCE LAST SAW DOCTOR ABOUT OWN HEALTH
AH75	TRIED TO FIND HEALTH INSURANCE ON OWN
AI28	HOW LONG SINCE LAST HAD HEALTH INS
AJ114	TIME SINCE LAST DOC VISIT FOR ROUTINE CHECK-UP
AJ136	NEEDED TO SEE MED SPECIALIST PAST YR
INS	CURRENTLY INSURED
ELIGPRG3	MEDI-CAL (MEDICAID)/HEALTHY FAM ELIG. -
CARE_PV	HAD A PREVENTIVE CARE VISIT IN PAST YEAR
DOCT_YR	VISITED DOCTOR IN PAST 12 MOS
USUAL	HAVE USUAL PLACE TO GO TO WHEN SICK OR NEEDING HEALTH ADVICE
USUAL_TP	USUAL SOURCE OF CARE (7 LVLS)
USOC	USUAL SOURCE OF CARE OTHER THAN ER
USUAL5TP	USUAL SOURCE OF CARE (5 LVLS)
HMO	HMO STATUS

Pink module from the weighted correlation network estimated during the first stage of Fuzzy Forests using data from 2013-2014 CHIS surveys. This module encompasses variables dealing with usual source of care, HMO indicator and current insurance status.

Supplementary Table 7: “Red” Fuzzy Forest Module

CHIS Feature	CHIS Feature Label
ACMDNUM	# OF DOCTOR VISITS PAST YEAR
AH102_P1	# NIGHTS IN HOSPITAL PAST 12 MOS
AH3_P1	KIND OF PLACE FOR USUAL SOURCE OF HEALTH CARE
AH34NEW	MOTHER BORN IN U.S.
AH35NEW	FATHER BORN IN U.S.
AH95_P1	# TIMES VISITED ER IN PAST 12 MOS
AI22A_P	NAME OF HEALTH PLAN
AI22C	MAIN HEALTH PLAN IS HMO
AI25	COVERED FOR PRESCRIPTION DRUGS
AI25NEW	RX COVERAGE EDITED FOR MEDI-CAL/HF
AJ102	SOUGHT APPNT W/DOC IN 2 DAYS PAST YR
AJ103	HOW OFTEN GET APPNT IN 2 DAYS
AJ106	USUAL PLACE FOR HLTH CARE CHANGED PAST YR
AJ112	HOW OFTEN DOC LISTENS CAREFULLY
AJ113	HOW OFTEN DOC CLEARLY EXPLAINS WHAT TO DO
AJ135	INSURANCE NOT ACCEPTED BY DOC PAST YR
AJ137	HAD TROUBLE FINDING SPECIALTY DR PAST 12 MOS
AJ138	NOT ACCEPTED AS NEW PATIENT BY SPECIALIST PAST YR
AJ139	INSURANCE NOT ACCEPTED BY SPECIALIST PAST YR
AJ77	HAVE PERSONAL DOCTOR AS MAIN MEDICAL PROVIDER
AJ78	CONTACT DOC’S OFFICE WITH MEDICAL QUESTION PAST 12 MOS
AJ79	HOW OFTEN GOT RESPONSE FROM DOC’S OFFICE
AM34	TELEPHONE CALLS RECEIVED
INS12M	MOS COVERED BY HEALTH PLANS LAST 12 MOS
INSANY	ANY INSURANCE IN LAST 12 MOS
OMBSRR_P1	OMB/CURRENT DOF RACE - ETHNICITY
PC_INS	PRIMARY CARE: HAVE DIFFICULTY FINDING A PROVIDER THAT ACCEPTS THEIR INSURANCE
PCTLF_P	PERCENT LIFE IN US
RACECN_P2	RACE - CENSUS 2000 DEFINITION
RACEDF_P1	FORMER DOF RACE - ETHNICITY
RACEHP2_P1	RACE - UCLA CHPR DEFINITION, UNABRIDGED
SC_INS	SPECIALTY CARE: DIFFICULTY FINDING PROVIDER ACCEPTS THEIR INSURANCE
SC_NEWP	SPECIALTY CARE: HAVE DIFFICULTY FINDING A PROVIDER THAT ACCEPTS NEW PATIENTS
SRH	SELF-REPORTED LATINO/HISPANIC
SRO	SELF-REPORTED OTHER RACE
TIMAPPT	ABLE TO GET AN APPOINTMENT IN A TIMELY WAY

Red module from the weighted correlation network estimated during the first stage of Fuzzy Forests using data from 2013-2014 CHIS surveys. This module encompasses variables dealing with usage of medical care and satisfaction with medical care provided and insurance difficulties as well as race.

Supplementary Table 8: “Turquoise” Fuzzy Forest Module

CHIS Feature	CHIS Feature Label
AB18	TAKING DAILY MEDICATION TO CONTROL ASTHMA
AB19	FREQUENCY OF ASTHMA SYMPTOMS IN PAST 12 MOS : CURRENT ASTHMATICS
AB40	STILL HAS ASTHMA
AB41	ASTHMA EPISODE/ATTACK IN PAST 12 MOS
AB43	HEALTH PROFESSIONAL EVER GAVE ASTHMA MANAGEMENT PLAN
AB98	HAVE WRITTEN COPY OF ASTHMA CARE PLAN
AC11	#TIMES SODA LAST MONTH
AC46	#OF TIMES DRANK SWEET FRUIT DRINKS PAST MONTH
AC49	STOP SMOKING 1 DAY OR LONGER PAST YEAR
AC50	THINK ABOUT QUIT SMOKING IN NEXT 6 MOS
AF63	FEEL NERVOUS WORST MONTH
AF64	FEEL HOPELESS WORST MONTH
AF65	FEEL RESTLESS WORST MONTH
AF66	FEEL DEPRESSED WORST MONTH
AF67	FEEL EVERYTHING AN EFFORT WORST MONTH
AF68	FEEL WORTHLESS WORST MONTH
AF69B	EMOTIONS INTERFERE W/WORK WORST MONTH
AB70B	EMOTIONS INTERFERE W/CHORES WORST MONTH
AF71B	EMOTIONS INTERFERE W/SOCIAL LIFE WORST MONTH
AF72B	EMOTIONS INTERFERE W/RELATIONSHIPS WORST MONTH
AH13A	ER/URGENT CRE VISIT FOR ASTHMA LAST 12 MOS : CURRENT ASTHMATICS
AJ1	INS COVERS TREATMENT FOR MNTL HEALTH PROBLEMS
AJ107	CHANGE USUAL PLACE DUE TO INSURANCE PLAN
AJ129	EVENTUALLY RECEIVED MED CARE THAT WAS DELAYED
AJ19	COST/NO INSUR DELAYED GETTING PRESCRIPTION
AJ20	COST/NO INSR DELAYED GETTING NEEDED CARE
AJ81	DELAYED PRESCRIPTION FOR ASTHMA
AJ9	MD SPOKE DIFFERENT LANGUAGE REASON WHY DIFFICULT TO UNDERSTAND
AK28	HOW OFTEN FEEL SAFE IN NEIGHBORHOOD
AL22	RECEIVING SOCIAL SECURITY DISABILITY INS
AM19	PEOPLE IN NEIGHBORHOOD WILLING TO HELP EACH OTHER
AM21	PEOPLE IN NEIGHBORHOOD CAN BE TRUSTED
AM35	NEIGHBORHOOD WATCHES OUT FOR CHILDREN’S SAFETY
AB106_P	ER/URGT CARE VISIT FOR ASTHMA PAST YR UNABLE TO SEE OWN DR
AE_SODA	# OF TIMES DRINKING SODA PER WEEK
ASTS	ASTHMA SYMPTOMS PAST 12 MOS: POP WITH ASTHMA
ASTYR	ASTHMA SYMPTOMS PAST 12 MOS: POP WITH ASTHMA
CHORES2	CHORE IMPAIRMENT PAST 12 MONTHS
DISTRESS	SERIOUS PSYCHOLOGICAL DISTRESS
FAMILY2	SERIOUS PSYCHOLOGICAL DISTRESS
WORK2	WORK IMPAIRMENT PAST 12 MONTHS
SOCIAL2	SOCIAL LIFE IMPAIRMENT PAST 12 MONTHS
NUMCIG	# OF CIGARETTES PER DAY
RN_FORGO	REASONS FORGONE NECESSARY CARE
DSTRSYR	SERIOUS PSYCHOLOGICAL DISTRESS FOR WORST MONTH PAST YR
AC31_P1	# TIMES FAST FOOD PAST WEEK
DSTRS_P1	SERIOUS PSYCHOLOGICAL DISTRESS
AM38_P1	MAIN REASON FOR LAST MOVE
AB108_P1	CONFIDENCE TO CONTROL AND MANAGE ASTHMA
AB42_P1	WORKDAYS MISSED DUE TO ASTHMA IN PAST 12 MOS
AD32_P1	# OF CIGARETTES PER DAY
AESODA_P1	# OF TIMES DRINKING SODA PER WEEK (RECODE)
AE16_P1	# OF CIGARETTES SMOKED PER DAY IN PAST 30 DAYS
AJ131_P1	MAIN REASON FOR DELAYING NEEDED CARE

Turquoise module from the weighted correlation network estimated during the first stage of Fuzzy Forests using data from 2013-2014 CHIS surveys. This module encompasses variables dealing with asthma, emotional health, psychological distress, neighborhood friendliness, and smoking habits.

Supplementary Table 9: "Yellow" Fuzzy Forest Module

CHIS Feature	CHIS Feature Label
AB110_P	ER FOR DIABETES BECAUSE UNABLE TO SEE OWN DR
AB113	HAVE WRITTEN COPY OF DIABETES CARE PLAN
AB115	VISITED ER FOR HEART DISEASE IN PAST 12 MOS
AB116_P1	ER VISIT FOR HEART DISEASE BECAUSE UNABLE TO SEE OWN DR
AB114_P1	CONFIDENCE TO CONTROL AND MANAGE DIABETES
AB117	ADMITTED TO HOSPITAL OVERNIGHT/LONGER FOR HEART DX
AB118	MEDICAL PROVIDERS DEVELOPED HEART DISEASE PLAN
AB119	HAVE WRITTEN COPY OF HEART DISEASE CARE
AB120_P1	CONFIDENCE TO CONTROL AND MANAGE HEART DISEASE
AB23_P1	AGE FIRST TOLD HAVE DIABETES
AB24	CURRENTLY TAKING INSULIN
AB25	CURRENTLY TAKING DIABETIC PILLS TO LOWER BLOOD sugar
AB26	# OF TIMES R/R'S FAMILY/R'S FRIEND CHECK BLOOD sugar
AB27_P1	# OF TIMES DOC CHECKED FOR HEMOGLOBIN A1C LAST yr
AB28_P1	# OF TIMES DOC CHECKED FEET FOR SORES LAST YR
AB30	CURRENTLY TAKING medication to control high blood pressure
AB51_P1	TYPE I OR TYPE II DIABETES (PUF 1 YR RECODE)
AB52	EVER TOLD HAVE HEART FAILURE/CONGESTIVE
AB63	LAST EYE EXAM DILATED PUPILS
AD37W	WALKED AT LEAST 10 MIN FOR TRANSPORT PAST 7 DAYS
AE15A	SMOKES CIGARETTES EVERYDAY, SOME DAYS OR NOT AT ALL
AH49	MEDICARE COVERAGE PROVIDED THROUGH HMO
AH50_P1	MEDICARE COVERAGE PROVIDED THROUGH HMO
AH52_P1	SIGN UP FOR MEDICARE HMO DIRECTLY OR OTHERWISE
AI4	COVERED BY MEDICARE SUPPL POLICY
AJ80	DOC'S OFFICE HELP COORDINATE CARE W/ OTHER MED SVCS
AK1	WORK STATUS LAST WEEK
AM37	CURRENTLY PAYING OFF MORTGAGE/HOME LOAN
BMI_P	BODY MASS INDEX (PUF RECODE)
DIABCK_P1	# OF TIMES CHECKING FOR GLUCOSE/SUGAR PER MONTH
ELDER_IDX	ELDERLY
INS65	TYPE OF CURRENT HEALTH COVERAGE SOURCE FOR ELDERLY 65+
INSPR	COVERED BY PLANS PURCHASED ON OWN
MARIT_45	MARITAL STATUS - AGE 45 AND OLDER
AB114_P1	CONFIDENCE TO CONTROL AND MANAGE DIABETES
RBMI	BMI DESCRIPTIVE
SERVED	LENGTH OF TIME SERVED IN ACTIVE DUTY
SRAGE_P1	SELF-REPORTED AGE
WEIGHK_P	WEIGHT: KG - UCLA
WGHTK_P	WEIGHT: KG
WGHTP_P	WEIGHT: LBS
WRKST_P1	WORKING STATUS

Yellow module from the weighted correlation network estimated during the first stage of Fuzzy Forests using data from 2013-2014 CHIS surveys. This module encompasses variables dealing with diabetes, medicare, and weight.

Supplementary Table 10: Data Sections in 2013-2014 CHIS survey

Module	Characteristic
Section A	Demographic Information, Part I
Section B	General Health Condition
Section C	Health Behaviors
Section D	General Health, Disability and Sexual Health
Section F	Mental Health
Section G	Demographic Information, Part II and Child Care
Section H	Health Insurance
Section J	Health Care Utilization and Access
Section K	Employment, Income, Poverty Status and Food Security
Section L	Public Program Participation
Section M	Housing and Community Involvement
Section N	Demographic Info Part II, Geographic
Section Q	Screening Information

Data sections from 2013-2014 CHIS surveys. Note that the weighted correlation network grouped the variables differently than the survey structure.
